# First clinical experience with posterior lumbar interbody fusion using a thermal-sprayed silver-containing hydroxyapatite-coated cage

**DOI:** 10.1186/s13018-023-03882-7

**Published:** 2023-05-30

**Authors:** Tadatsugu Morimoto, Masatsugu Tsukamoto, Katsuhiro Aita, Nobuyuki Fujita, Masaaki Mawatari

**Affiliations:** 1 Department of Orthopedic Surgery, Faculty of Medicine, Saga University, Nabeshima 5-1-1, Saga, Japan; 2 Department of Orthopedic Surgery, Saga Memorial Hospital, Saga, Japan; 3 Department of Orthopedic Surgery, Faculty of Medicine, Fujita Health University, Aichi, Japan

**Keywords:** Orthopedic fixation devices, Spinal diseases, Surgical wound infection, Hydroxyapatite, Silver, Spinal fusion

## Abstract

**Background:**

To investigate the possibility of silver (Ag)-induced adverse events and the degree of bone fusion in posterior lumbar interbody fusion surgery using an Ag-containing hydroxyapatite (HA) lumbar interbody cage.

**Methods:**

An Ag-HA cage consisting of highly osteoconductive HA interfused with Ag was developed, and we applied it clinically at three university-affiliated hospitals from April 2020 to December 2020. During the 12-month postoperative observation period, Ag-related adverse events, neuropathy, and postoperative complications were investigated as indicators of safety, while clinical improvement and the fusion status were investigated as indicators of efficacy. Clinical improvement was defined as improvement beyond the minimum clinically important difference (MCID) in the numerical rating scale (NRS; 1.6) for low back and lower limb pain and the Oswestry Disability Index (ODI; 12.8).

**Results:**

We performed lumbar interbody fusion using an Ag-HA cage for 48 patients (female, n = 25; mean age, 67.5 years). The mean preoperative NRS was 6.4 (standard deviation, 1.9), while the mean preoperative ODI was 44 [12]. No adverse effects (i.e., argyria) were identified during the 12-month observation period. Surgical site infection occurred in one case, although the implant was preserved via immediate debridement. In total, 39 (81%) participants showed clinical improvement beyond MCID for both NRS and ODI. Bone fusion was achieved at 45 levels (88%) at 6 months and 48 levels (91%) at 12 months postoperatively.

**Conclusions:**

The results of this study suggest that Ag-HA cages can be safely used in spinal fusion procedures and have the potential to prevent postoperative infections, prevent deterioration of the quality of life, and result in favorable outcomes. Larger-scale and longer-term follow-up studies will be required to corroborate these conclusions.

*Trial registration* UMIN 000039964 (date: April 01, 2020).

## Background

Posterior lumbar interbody fusion (PLIF) and transforaminal lumbar interbody fusion (TLIF) are established treatments for various pathologies of the lumbar spine (i.e., degenerative pathologies, trauma, infection, and tumors) [1]. With the increase in the aging population, the number of patients with high surgical risk, including those with osteoporosis, comorbidities, and/or compromised immunity, has also increased [2]. Non-fusion and surgical site infection (SSI) are well-known complications, particularly among older patients who undergo PLIF or TLIF. Non-fusion is a relatively common complication after PLIF or TLIF, with an incidence of 0–35% [3]. The incidence of SSI requiring revision surgery is reportedly 2% [4].

Surface coating technologies represent a strategy to address these complications because they improve osteoconductivity and provide antibacterial properties. Several antibacterial coatings have been developed for orthopedic implants [5]. Notable products include silver (Ag)-coated megaprostheses and antibiotic coatings [5, 6]. Ag is a well-established coating used for various medical materials (e.g., megaprostheses, vascular and urinary catheters, dressing materials, vascular prostheses, bone cement, suture material, skin dressings, contact lenses, heart valves, and pins for external fixation) because it shows broad-spectrum antibacterial activity, is less resistant than are antibiotics, inhibits biofilm formation, has long-lasting effects, and shows low toxicity in the human body [5–7]. Ag at high concentrations in vitro can be toxic to osteoblasts, although toxicity has not been observed at low concentrations [5, 8]. Therefore, antimicrobial and osteogenic properties could be obtained by conditioning the concentration of Ag. Additionally, hydroxyapatite (HA) is biocompatible and osteoconductive, and it can facilitate the deposition of bone on an implant surface and form a direct chemical bond between the bone and implant surface (osseointegration) without involving soft tissue [9, 10]. HA coatings have been shown to promote osseointegration of dental and orthopedic implants (i.e., pedicle screw and hip prosthesis) [10–12].

Thus, to simultaneously achieve antimicrobial and osteogenic properties, we developed Ag-containing HA (Ag-HA) by interfacing osteoconductive HA with antibacterial Ag [8, 13]. After it was established that a 3% Ag-HA coating has adequate biocompatibility and low toxicity in vitro and in vivo [14–16], this technology was applied to implants to create a cementless 3% Ag-HA-coated hip system for total hip arthroplasty (THA). However, cytotoxicity, including osteoblast, liver damage, nephropathy, neuropathy, leukopenia, and argyria, has been reported at high concentrations of Ag [6, 8]. Argyria is a typical side reaction of Ag that is sometimes severely disfiguring with blue-gray skin discoloration due to Ag precipitation [6, 8].

No postoperative infections or adverse reactions associated with the use of Ag have been observed [15, 17]. Based on extensive experiments in animal models and the clinical success of the AG-HA coating in THA, 3% Ag-HA-coated titanium cages (Ag-HA cages) for PLIF were developed to prevent postoperative spinal implant infection and enhance fusion ability. In April 2020, these were successfully commercialized (ResitageTM, Kyocera, Kyoto), and clinical applications was initiated [7].

To evaluate the appropriateness of further large-scale trials comparing Ag-HA cages with conventional cages and to obtain information on the basis of the study protocol, we first performed a multicenter pilot study wherein we investigated the possibility of Ag-induced adverse events and the degree of bone fusion in PLIF surgery using an Ag-containing HA lumbar interbody cage.

## Methods

### Study design and setting

This multicenter, single-arm, prospective study evaluated the safety and osteoconductivity of the Ag-HA-coated intervertebral cage used in PLIF or TLIF. The study was approved by the institutional review board of our institution (#2019-07-R-11) and has been registered in the University Hospital Medical Information Network clinical trials registry (UMIN 000039964). Moreover, it adhered to the principles of the Declaration of Helsinki. After receiving full approval from the local ethical committee, participants were recruited from three university-affiliated hospitals between April 2020 and December 2020. Written informed consent was obtained from all participants.

### Study participants

The inclusion criteria were as follows: (1) age ≥ 20 years; (2) ≥ grade II lumbar degenerative spondylolisthesis or lumbar spinal canal stenosis with local coronal imbalance (≥ 5 mm transverse vertebral translation and/or ≥ 5° lateral disc wedging angle) necessitating single or dual-level TLIF or PLIF as determined by the physician in charge at each facility; (3) no history of metabolic bone disease, bone tumors, or cancer metastasis; and (4) ≤ 2 fused vertebrae. The exclusion criteria were as follows: (1) known allergy or hypersensitivity to Ag based on the patient’s medical history, (2) presence of general inflammatory disease or osteoporosis (bone mineral density ≤ 70% of the Young Adult Mean percentage), and (3) a history of lumbar surgery.

### Intervention

**Fig. 1:**
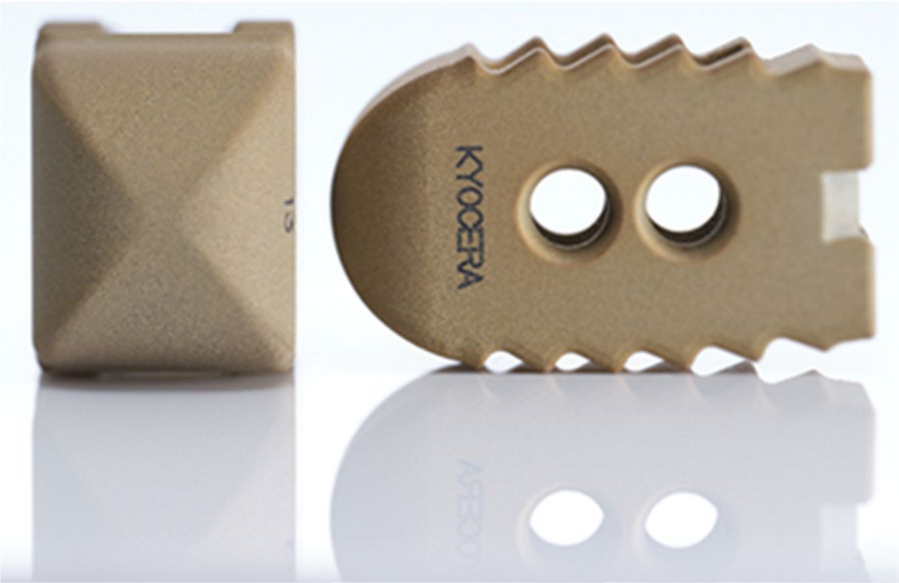
Ag-HA-coated titanium cage. *Ag-HA* silver-containing hydroxyapatite

A cage made of titanium alloy with a design profile conforming to the anatomical shapes was used as the base material. The surface of the cage that faced the bone was coated with HA. Ag-HA was prepared by adding Ag_2_O powder to HA powder (KYOCERA, Kyoto, Japan). Ag-HA was thermal sprayed as a coating material for the creation of an Ag-HA-coated titanium cage (Ag-HA coating thickness: 2 mm) (Fig. 1).

Patients underwent a PLIF or TLIF procedure with open or percutaneous pedicle screws, bone graft (morselized local bone during decompression, allograft, demineralized bone matrix [Grafton™; Medtronic Sofamor, Danek, Minneapolis], or a combination of these), and an Ag-HA-coated cage. Local bone grafts were obtained from the spinous processes, lamina, and facets. One or two interbody cages filled with bone graft were inserted into the interbody space. In standard cases, two cages were used; however, in cases of degenerative scoliosis and revision surgery, a single cage was considered, with the final decision left to the surgeon. After the screws were inserted, rods were placed on both sides, and moderate pressure was applied posteriorly to prevent cage deviation and achieve proper local lumbar kyphosis. Postoperatively, all patients wore a soft lumbar corset for 3 months and underwent standardized physical therapy, including exercises for strengthening the trunk and extremity muscles and walking.

### Data collection

Data on participants’ demographic and clinical characteristics, such as age at surgery, sex, comorbidities, indications for surgery, and the PLIF or TLIF segment level, were obtained from medical and surgical records.

The primary outcome of interest was Ag-induced adverse events. During the postoperative follow-up, each patient was monitored for adverse reactions to Ag (systemic/local argyria, delayed wound healing, and neurological symptoms) by certified spine surgeons at each facility. Systemic/local argyria was evaluated based on the presence of ash-colored skin, which can occur in argyria (systemic Ag intoxication). Local argyria was assessed for the skin around the surgical wound, and systemic argyria was assessed for the skin all over the body. Delayed wound healing was defined as the lack of wound healing at more than 10 days after surgery. Neurological symptoms were evaluated via medical examinations. Neurological symptoms that appeared for the first time after surgery and could not be explained organically after various examinations, including imaging studies, were defined as neurological symptoms due to Ag toxicity. In addition, postoperative complications were examined.

Effectiveness indicators included clinical improvement and fusion status. The numerical rating scale (NRS) for low back pain (LBP)/lower limb pain and Oswestry Disability Index (ODI) scores [18] were evaluated as measures of clinical improvement. Intervertebral bone fusion and segmental instability were evaluated as measures of the fusion status. These effectiveness indicators were assessed preoperatively and at 6 and 12 months after surgery. Imaging parameters were assessed by two independent certified spine surgeons using lateral dynamic X-rays and multidetector-row computed tomography (MDCT). Intervertebral bone fusion was defined as completing the following conditions: (1) osseous continuity between the bony endplate and implant on both coronal and sagittal MDCT images and (2) less than 3° motion on flexion–extension [3, 19]. Additionally, the presence of a visible gap around the pedicle screws, the presence of cage subsidence, and vertebral endplate cyst formation (VECF) were investigated as imaging indicators related to segmental instability. Cage migration of > 2 mm into the vertebral endplate was defined as cage subsidence [19]. VECF positivity was defined as an endplate cyst that appeared de novo or was larger at each time point than it was at the preoperative assessment [20].

### Statistical analysis

Baseline characteristics and outcomes were reported for the overall population. Normally and non-normally distributed continuous variables are reported as mean (standard deviation [SD]) and median (interquartile range [IQR]), respectively. Categorical variables are presented as the frequency (percentage). For the effectiveness measures, NRS and ODI, the percentage of participants who achieved an improvement beyond the minimum clinically important difference (MCID; 1.6 for NRS and 12.8 for ODI) was also reported [21]. Inter- and intra-observer agreements regarding the imaging-based improvement were evaluated by calculating kappa (κ) values. All data were managed using Stata version 17 (StataCorp LLC; College Station, TX, USA).

## Results

**Fig. 2:**
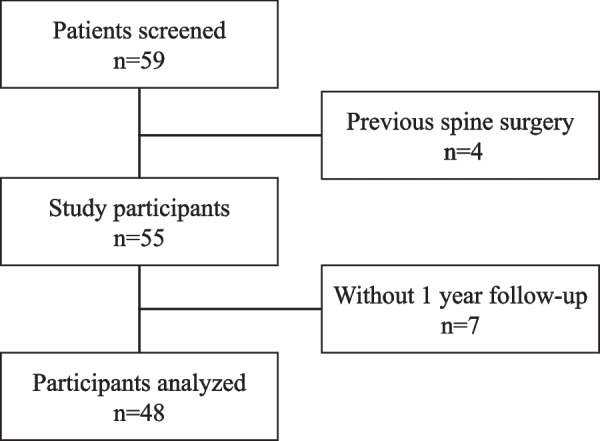
Study flowchart

Figure 2 shows a flowchart of the present study. Of the 59 patients who were eligible to participate, 55 were included in the study, excluding four patients with a history of spinal surgery. Of these, 48 participants who could be followed up for 1 year were analyzed. The demographic and clinical data of the patients are summarized in Table 1. The participants included 25 (52%) women, and the median age was 69 (IQR: 62.5–73). Surgery was performed for the following conditions: spinal canal stenosis (n = 20), degenerative spondylolisthesis (n = 20), disc hernia (n = 5), and spondylolysis (n = 3). Immunocompromise was suspected in 12 participants (e.g., diabetes mellitus, rheumatoid arthritis, or steroid use). Forty-five participants underwent fixation of one level, and three participants underwent fixation of two levels; thus, a total of 51 intervertebral spaces were analyzed. Thirty-four intervertebral spaces (L2–3: 1, L3–4: 11, L4–L5: 16, L5–S1: 6) requiring single Ag-HA cage insertion and 17 intervertebral spaces (L2–3: 0, L3–4: 3, L4–L5: 6, and L5–S1: 8) requiring dual Ag-HA cage insertion were included in the study.

**Table 1:** Demographic and clinical data of the participants

	n = 48
Age, year	69 (62.5–73)
Sex (female)	25 (52)
Body mass index, kg/m^2^	24.7 (3.2)
Comorbidity	
Diabetes mellitus	8 (17)
Cardiovascular diseases	4 (8.3)
Rheumatoid arthritis	4 (8.3)
Steroid use	4 (8.3)
Indication for surgery	
Spinal canal stenosis	20 (42)
Degenerative spondylolisthesis	20 (42)
Spondylolysis	3 (6.3)
Disc hernia	5 (10)
Level*	
L2/3	1 (2.1)
L3/4	14 (27)
L4/5	22 (43)
L5/S	14 (27)
Dual cage*	17 (33)

Age is presented as the median (IQR),while categorical variables are presented as the frequency (percentage)

^*^Number of intervertebral spaces fixed, not the number of participants; thus, n = 51

### Adverse events

During the postoperative follow-up period, no participant showed any signs of systemic and/or local argyria or neurological symptoms due to Ag toxicity. However, a 58-year-old man with no underlying disease developed SSI (deep infection). The participant was reoperated for debridement as soon as possible and treated with antibiotics postoperatively. After the reoperation, the infection was quiescent and did not require implant removal. Another patient developed pain in the left leg and a disc herniation at the L3/4 level 3 months after L4/5/S1 PLIF. Fusion extension surgery was performed at 5 months after the first surgery.

### Effectiveness indicators

The results of clinical improvement and fusion status are summarized in Table 2. In terms of clinical improvement at 12 months postoperatively, 81% of participants had achieved an improvement over MCID in both NRS and ODI. Intervertebral bone fusion was achieved by 88% of participants at 6 months postoperatively and 91% at 12 months postoperatively. Visible gap around the pedicle screws and cage subsidence were both limited to a few participants, and VECF was 29% at 6 months and 22% at 12 months. The kappa values for intervertebral bone fusion, cage subsidence, and VECF were 0.85 (95% confidence interval (CI): 0.57–1.13), 1 (95% CI 1–1), and 0.73 (95% CI 0.38–1.08) for inter-observer agreement and 0.83 (95% CI 0.49–1.16), 1 (95% CI 1–1), and 0.83 (95% CI 0.49–1.16) for intra-observer agreement, respectively.

**Table 2:** Summary of clinical improvement and fusion status

	n = 48		
	Preoperative	6 months	12 months
Clinical improvement			
NRS for low back/lower limb pain	7 (5–8)	2 (1–3)*	1.5 (0.0–3.25)
Improvement over MCID (1.6)		37 (82)*	39 (81)
Oswestry Disability Index	44 (12)	12 (6–26)**	11 (3.5–29)
Improvement over MCID (12.8)		39 (83)**	39 (81)
Fusion status^†^			
Intervertebral bone fusion		42 (88)	44 (91)
Visible gap around the pedicle screws		2 (3.9)	4 (7.8)
Cage subsidence (> 2 mm)		3 (5.9)	3 (5.9)
Vertebral endplate cyst formation		15 (29)	11 (22)

Normally and non-normally distributed continuous variables are presented as the mean (standard deviation) and median (interquartile range (IQR)), respectively, while categorical variables are presented as the frequency (percentage)

*NRS* numerical rating scale, *MCID* minimum clinically important difference

^*^n = 45 because of three missing measurements

^**^n = 47 because of one missing measurement

^†^The number of intervertebral spaces fixed, not the number of participants; thus, n = 51

### Representative case

**Fig. 3:**
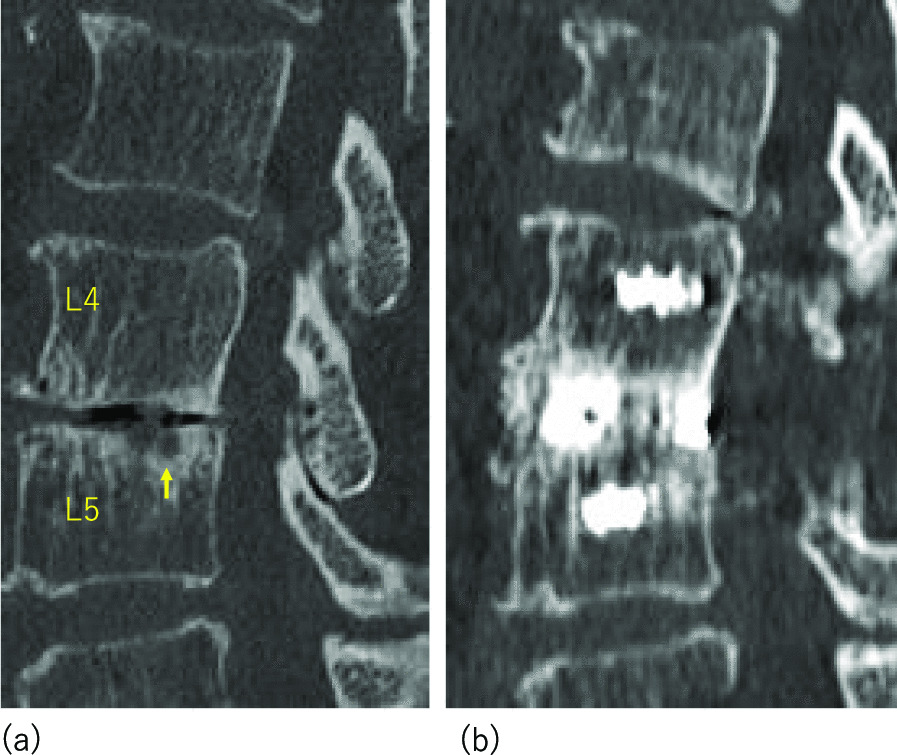
Representative case of an 81-year-old woman with stenosis of the lumbar spinal canal. **a** Preoperative sagittal lumbar computed tomography (CT) shows vertebral endplate cyst formation (VECF) (↑arrow) in the upper endplate of the L5 vertebra. **b** Sagittal lumbar CT performed 6 months after L4/5 posterior fusion shows intervertebral bony fusion, no halation around the cage, and complete VECF resolution

An 81-year-old woman with lumbar spinal canal stenosis that caused LBP and leg pain underwent PLIF using an Ag-HA cage. Six months after surgery, there was no LBP or metal artifact around the cage, and intervertebral fusion was achieved. Moreover, the VECF that existed preoperatively was completely resolved (Fig. 3).

## Discussion

In this study, we observed 48 participants who underwent TLIF or PLIF using Ag-HA-coated interbody cages and collected information regarding the appropriateness of cage use. Of the 48 participants (51 intervertebral levels), only one experienced SSI, and no Ag-induced complications occurred. In addition, > 80% of participants showed clinically meaningful symptomatic improvement at 12 months postoperatively, and the fusion rate was 91%, which is clinically acceptable. The results of this study could provide valuable information for conducting subsequent clinical trials comparing Ag-HA cages with conventional cages.

To the best of our knowledge, this is the first trial to evaluate the safety and efficacy of the Ag-HA cage for lumbar interbody fusion in patients with lumbar spine disease. Although Ag has antibacterial activity, it is also associated with adverse effects such as cytotoxicity or poor cytocompatibility [22]. The antibacterial mechanism of action of Ag particles includes binding to the thiol groups of enzymes, cell membranes, and nucleic acids, which results in structural abnormalities, damage to cell membranes, and inhibition of cell division [23–25]. These multifunctional actions of Ag on different intercellular targets make it difficult for bacterial strains to develop resistance. Ag-coated megaprostheses have been used in clinical practice; however, high concentrations of Ag were demonstrated to be toxic to osteoblasts, inhibiting ossification and contributing to osteolysis and postoperative loosening of the prosthesis [26]. Because the cytotoxic effect of Ag appears to be dose-dependent, it is important to control the concentration of compounding materials to achieve optimal antibacterial and osteogenic properties simultaneously [22, 27]. Low concentrations of Ag were found to have no cytotoxic effects on osteoblasts in vitro [22, 27]. We developed Ag-HA by combining 3% Ag with HA, which is known to have high osteoconductivity, and demonstrated that 3% Ag-HA is a safe material [14, 16] with good osteoconductivity [8, 28] and antibacterial activity [13, 29, 30] that can be used in vitro, in vivo, and in humans. Moreover, previous studies have demonstrated that the use of 3% Ag-HA-coated implants in THA markedly improved activities of daily living without causing any adverse reactions attributable to Ag in the human body [15, 17]. After favorable in vitro and in vivo results were obtained, we conducted this clinical trial using Ag-HA cages in PLIF or TLIF. Most reports on antimicrobial implants in orthopedic surgery have involved limb fractures and bone tumor reconstructions, and there have been few reports of antimicrobial implants used in the spine [7].

### Safety of the Ag-HA cage

Ag-related adverse reactions, such as argyria and mental or neurological disorders [31], hepatic and renal dysfunction [32], cytotoxicity [33], and mutagenicity [34], can result from a total dose of 4 g of Ag or a blood Ag concentration of ≥ 300 ppb [23, 32]. Regarding neurological damage, Seçinti et al. reported that the implantation of 23 g of Ag does not cause neuropathy, based on the listed dental literature [35]. The maximum amount of Ag contained in the Ag-HA implant for THA and the double cage was reported to be 3 mg [15] and 1.6 mg, respectively. In addition, after the insertion of the Ag-HA implant for THA, the blood Ag level was found to remain within the normal range (< 15 ng/mL), and the highest blood Ag level was 6.0 ng/mL [15]. Thus, in patients treated with the Ag-HA cage, the probability of developing argyria or other adverse reactions is considered extremely low, as shown in the present study. This is because the amount of Ag in the Ag-HA cage is much lower than that in the Ag-HA implant for THA, which has not been associated with any adverse events. In this study, no participant showed any signs of systemic and/or local argyria or neurological symptoms during the follow-up period. Thereafter, no patients showed any sign of wound dehiscence, systemic and/or local argyria, or neurological symptoms that worsened during the follow-up period. Implant failure did not occur in any of the patients.

In this study, a 58-year-old man with no underlying disease developed a deep infection. In the case of deep SSI in instrumented spine surgery, biofilm formation on the instrument is a major factor in the severity and refractoriness of the infection. In such cases, the removal of the instrument is frequently required. Although infection of the cage was considered in this case, the cage was not removed at the first revision surgery because of the expected effect of Ag-HA in inhibiting biofilm formation [36]. It may have been fortunate that the infection was cured with only the first reoperation, or it may have been due to the significant effect of Ag-HA. These possibilities would need to be confirmed in future large-scale studies.

### Efficacy of the Ag-HA cage

The efficacy of the Ag-HA cage was evaluated based on clinical and radiological assessments. With regard to clinical findings that are effectiveness indicators, all patients showed an improvement in the NRS score for LBP and lower limb pain and the ODI score for LBP-related quality of life. In a systematic review of the quantitative evaluation of bony fusion in lumbar interbody fusion, Formica et al. [3] analyzed 67 articles, of which 31, 19, and 17 articles used X-ray, computed tomography (CT), and both. The review recommended that CT is the most effective method for assessing instability, whereas lateral dynamic X-rays alone are limited because they tend to produce false-negative results and a high rate of bone fusion. This study evaluated segmental instability and radiolucency or a gap around the implant using lateral dynamic X-rays and MDCT 12 months postoperatively. Previous reports that described the fusion rate in PLIF or TLIF were used to assess patients using lateral dynamic X-rays and MDCT, and the fusion rate ranged from 65 to 100% [3]. One of the main reasons for this wide variation in interbody fusion rate could be the insufficient common criteria for assessing arthrodesis [3, 16, 37]. In a systematic review by Formica et al., the mean bone fusion rates for PLIF (26 papers, 1591 patients) and TLIF (21 papers, 1819 patients) were 93% (95% CI 90–95; χ^2^: 64.4, degree of freedom (df): 25, p < 0.001; I^2^: 61.2%; τ^2^: 0.03) and 94% (95% CI 91–97; χ^2^ = 99.2, df: 20, p < 0.001; I^2^: 79.8%; τ^2^: 0.06) [3]. The fusion rate in patients in whom the Ag-HA cage was used in this study was 91% for all intervertebral spaces at 12 months after surgery; this rate appears acceptable. The fusion rate in patients in whom the Ag-HA cage was used in this study was 91% for all intervertebral spaces at 12 months after surgery; this rate seems acceptable. In PLIF and TLIF, in addition to intervertebral bone fusion, biological fixation of the bone and cage are very important. Intramedullary Ag-HA implants placed in the lower extremities reportedly showed good bone formation and osseointegration in rats and humans [15, 17, 30], although these have not been examined in the lumbar intervertebral space, which has poorer bone fusion conditions than those in the lower extremity marrow because of the difference in blood flow and the contact area between the bone and implant [38].

Recently, it has been reported that VECF after lumbar interbody fusion using a cage in the early postoperative period may be a predictor of pseudoarthrosis or non-fusion [39], and the relationship between VECF and non-fusion has been investigated for different types of interbody cages, including polyetheretherketone (PEEK), titanium, titanium-coated PEEK, and porous tantalum; however, no studies have investigated this relationship in patients treated using Ag-HA cages [39, 40]. In the present study, cage subsidence occurred in three cases (5.9%) at 12 months after surgery. The modulus of elasticity of the Ag-HA cage, which is made of titanium coated with Ag-HA, is the same as that of titanium and higher than that of bone. Although it has been hypothesized that a high modulus of elasticity leads to increased rates of subsidence [41], this was only observed in three cases in our study population. Cage subsidence may be prevented by adequate bone grafting and careful procedures that do not destroy the bony endplate. Regarding the suitability of VECF as an indicator of bone fusion, at 12 months postoperatively, we noted VECF incidences of 13.7% to 60% in the PEEK cage group, 0–17.3% in the titanium cage group, and 21.6% in the Ag-HA cage group. Thus, the PEEK cage tends to be associated with a higher incidence of VECF [39, 40], which seems true for the Ag-HA and titanium cages. The respective rates of VECF and bone fusion were 29% and 88% at 6 months after surgery and 22% and 91% at 12 months after surgery, respectively. Therefore, we speculated that the Ag-HA cage did not interfere with bone fusion and that VECF disappeared due to bone remodeling caused by reduced micromovement in cases where the bone fusion between the Ag-HA cage and the endplate progressed [42].

### Limitations

The present study was associated with some limitations. First, the lack of a control group made it impossible to evaluate the superiority of AG-HA cages over conventional cages in terms of antimicrobial resistance and fusion rate. This research question should be addressed in subsequent clinical trials based on the results of this study. In addition, the safety of the Ag-HA cage may not have been adequately confirmed because of the small sample size and short follow-up period. This study population needs to be closely monitored, and further cases need to be accumulated. Therefore, a prospective multicenter clinical trial (UMIN 000039964) is currently underway.

## Conclusions

We developed an Ag-HA-coated cage, which has both the antibacterial activity of Ag and the osteoconductive activity of HA, for spinal fusion. The clinical trial was successful, with no cases of Ag-induced adverse effects and acceptable clinical results. The Ag-HA cage has the potential to reduce postoperative infections, prevent deterioration of the quality of life, and result in favorable outcomes in patients undergoing PLIF and TLIF. Larger-scale and longer-term follow-up studies will be required to corroborate these conclusions.

## Data Availability

The datasets used and/or analyzed during the current study are available from the corresponding author on reasonable request.

## Abbreviations

PLIF: Posterior lumbar interbody fusion
TLIF: Transforaminal lumbar interbody fusion
SSI: Surgical site infection
Ag: Silver
HA: Hydroxyapatite
THA: Total hip arthroplasty
NRS: Numerical rating scale
LBP: Low back pain
ODI: Oswestry disability index
MDCT: Multidetector-row computed tomography
VECF: Vertebral endplate cyst formation
SD: Standard deviation
PEEK: Polyetheretherketone
